# tRNAs Promote Nuclear Import of HIV-1 Intracellular Reverse Transcription Complexes 

**DOI:** 10.1371/journal.pbio.0040332

**Published:** 2006-10-03

**Authors:** Lyubov Zaitseva, Richard Myers, Ariberto Fassati

**Affiliations:** 1 Wohl Virion Centre, London, United Kingdom; 2 Medical Research Council Centre for Medical Molecular Virology, Division of Infection and Immunity, University College London, London, United Kingdom; 3 Centre of Postgenomic Virology, Division of Infection and Immunity, University College London, London, United Kingdom; Fred Hutchinson Cancer Research Center, United States of America

## Abstract

Infection of non-dividing cells is a biological property of HIV-1 crucial for virus transmission and AIDS pathogenesis. This property depends on nuclear import of the intracellular reverse transcription and pre-integration complexes (RTCs/PICs). To identify cellular factors involved in nuclear import of HIV-1 RTCs, cytosolic extracts were fractionated by chromatography and import activity examined by the nuclear import assay. A near-homogeneous fraction was obtained, which was active in inducing nuclear import of purified and labeled RTCs. The active fraction contained tRNAs, mostly with defective 3′ CCA ends. Such tRNAs promoted HIV-1 RTC nuclear import when synthesized in vitro. Active tRNAs were incorporated into and recovered from virus particles. Mutational analyses indicated that the anticodon loop mediated binding to the viral complex whereas the T-arm may interact with cellular factors involved in nuclear import. These tRNA species efficiently accumulated into the nucleus on their own in a energy- and temperature-dependent way. An HIV-1 mutant containing MLV *gag* did not incorporate tRNA species capable of inducing HIV-1 RTC nuclear import and failed to infect cell cycle–arrested cells. Here we provide evidence that at least some tRNA species can be imported into the nucleus of human cells and promote HIV-1 nuclear import.

## Abstract 2

HIV-1 reverse transcription and pre-integration complexes (RTC) are imported into the nucleus, where they integrate into the cellular DNA; some tRNA species undergo nuclear re-uptake and promote HIV RTC nuclear import.

## Introduction

HIV-1 infects cells of the immune system expressing CD4, in particular T helper lymphocytes and macrophages [1]. Studies on the acute phase of SIV (simian immunodeficiency virus) and HIV infection in primate models and in patients have indicated that infection of mucosal resting CD4^+^ memory T cells and macrophages is crucial for subsequent viral spread and progression to AIDS [2–5]. Moreover, HIV-1 infection of microglial cells in the central nervous system is a necessary step for the development of AIDS-related dementia [6]. Macrophages, microglial cells, and unstimulated CD4^+^ memory T lymphocytes do not undergo mitotic division, underscoring HIV-1 and other lentiviruses' ability to infect non-dividing cells [7,8].

HIV-1 infection of non-dividing cells is thus recognized as having a fundamental role in virus transmission as well as AIDS pathogenesis, and it has been extensively investigated. Biochemical studies have indicated that infection of non-dividing cells depends on active nuclear import of the intracellular viral complex mediating reverse transcription and integration of the viral genome (hereafter called reverse transcription complex or RTC) [9].

Several components of the HIV-1 RTC are implicated in its nuclear import, but little is known of the cellular pathways involved (reviewed in [10,11]). Although the identification of nuclear localizing signals (NLS) within the RTC seemed the most obvious step to understand HIV-1 nuclear import, several limitations of this approach have now become apparent. First, mutations of viral proteins may have pleiotropic effects or compromise virus viability, which confound phenotypic analysis. Second, multiple pathways may exist for nuclear import of HIV-1 RTCs, and deletion or mutation of one or more viral components may not result in a detectable phenotype. Third, cellular factors present in the RTC may also be involved [12]. Lastly, rapid virus uncoating may be critical to let the RTC become engaged with the cellular nuclear import machinery, since murine leukaemia virus (MLV) capsid protein, and presumably the presence of an intact core, has been shown to have a negative impact on infection of non-dividing cells [13].

We have developed a different experimental approach that primarily aims to identify the components of the cellular machinery responsible for nuclear import of HIV-1 RTCs [14]. This approach is based on the use of purified RTCs as substrate in the nuclear import assay, which is well established in the nuclear import field and has been applied to study nuclear import of several viruses (reviewed in [15–17]). Briefly, cells are treated with digitonin to permeabilize selectively the plasma membrane, leaving intact the nuclear envelope. Cytosolic contents are washed out, and nuclear import is reconstituted by the addition of fluorescent-labelled purified RTCs, cytosolic extracts or purified transport receptors, the components of the Ran system, and an energy-regenerating system. Nuclear accumulation of the substrate is then analyzed by fluorescence microscopy [18]. This approach circumvents several of the problems listed above. First, it does not depend on mutations of viral proteins, and second, putative nuclear import components can be tested individually, thus multiple and potentially redundant import pathways can be identified and dissected.

Using this approach we have recently found that importin 7 (imp7) is implicated in nuclear import of HIV-1 RTCs and that siRNA-mediated depletion of imp7 inhibits HIV-1 infection [14]. However, knock down of imp7 reduces HIV-1 infection by only a few fold, and it has been suggested that imp7 alone may not be sufficient for HIV-1 infection in primary macrophages [19], which has led to the hypothesis that additional factors might be involved. To identify additional pathways for HIV-1 nuclear import, we have fractionated cytosolic extracts by ammonium sulphate (AS) precipitation and chromatography and followed RTC import activity using the nuclear import assay. The active fraction was purified to near-homogeneity, and surprisingly, it turned out to contain tRNAs. Such tRNAs induced HIV RTC nuclear import, were incorporated into virus particles, and accumulated into the nucleus on their own in an energy- and temperature-dependent way.

## Results

**Figure 1:**
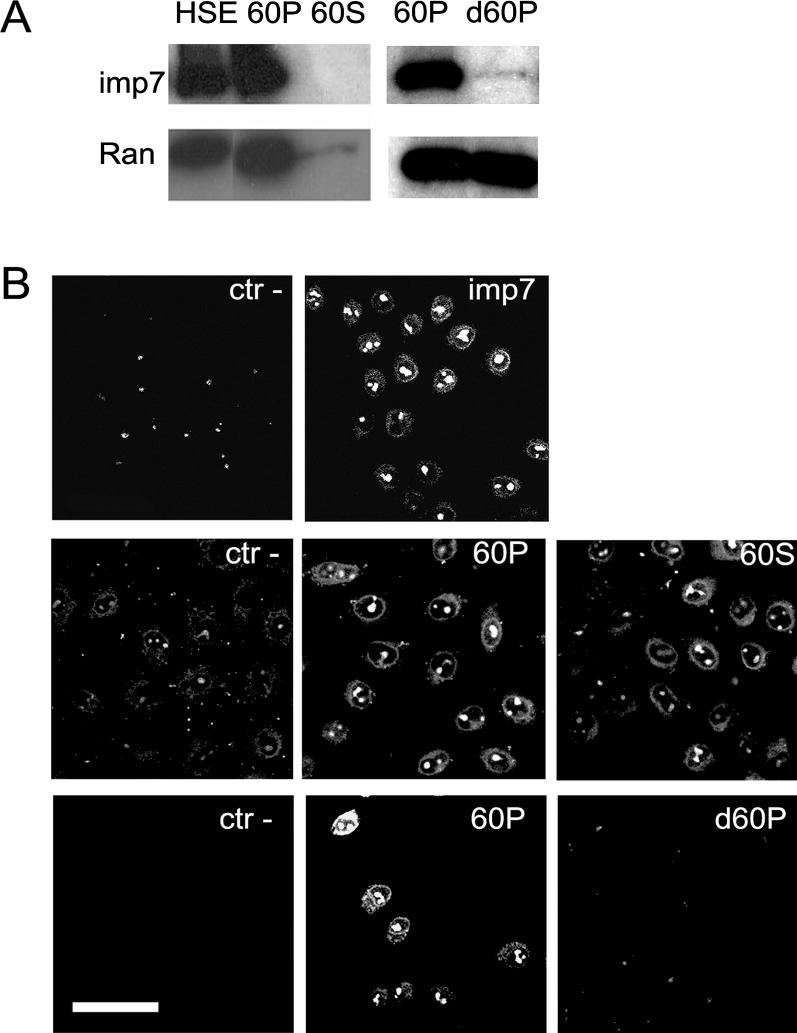
imp7 and Additional Cytosolic Factors Support RTC Nuclear Import (A) Western blot showing depletion of both imp7 and Ran in the supernatant (60S) of the 60% AS precipitation step and depletion of imp7 but not Ran from the 60% AS precipitation pellet (60P) after low-substitution Phenyl-Sepharose chromatography (d60P). (B) Nuclear import in permeabilized HeLa cells in the presence of labelled RTCs, 1× energy-regenerating system and buffer (panel ctr−), 1μM imp7 + 1× Ran mix (panel imp7), 0.5 mg/ml of the pellet (panel 60P) or supernatant (panel 60S) fractions from the 60% AS precipitation step, or 60P after low-substitution Phenyl-Sepharose chromatography (panel d60P). Images were acquired by confocal microscopy using the same settings. Scale bar indicates 25 μm.

To investigate whether there are other cellular factors, which in addition to imp7 and Ran can induce nuclear import of HIV-1 RTCs, we have fractionated high-speed cytosolic extracts (HSE) from HeLa cells [20] by AS precipitation. Preliminary experiments showed that imp7 and other importins precipitated in 45% AS and Ran precipitated in 60% AS (unpublished data). We therefore subjected HSE to a 60% AS precipitation step and tested the resulting pellet and supernatant in the nuclear import assay. Although imp7 and Ran were found in the 60% AS pellet fraction only (Figure 1A), both pellet (60P) and supernatant (60S) from this precipitation step supported RTC nuclear import equally well (Figure 1B). Incubating the 60P fraction with a low-substitution Phenyl-Sepharose column, a procedure that depletes all importins from HSE [21] resulted in the loss of imp7 (Figure 1A) and RTC nuclear import activity from the 60P fraction (Figure 1B). These results showed that additional RTC nuclear import factors were present in HSE, which were not precipitated in 60% AS and had properties distinct from imp7 and other importins.

### Characterization of the 60S Fraction

**Figure 2:**
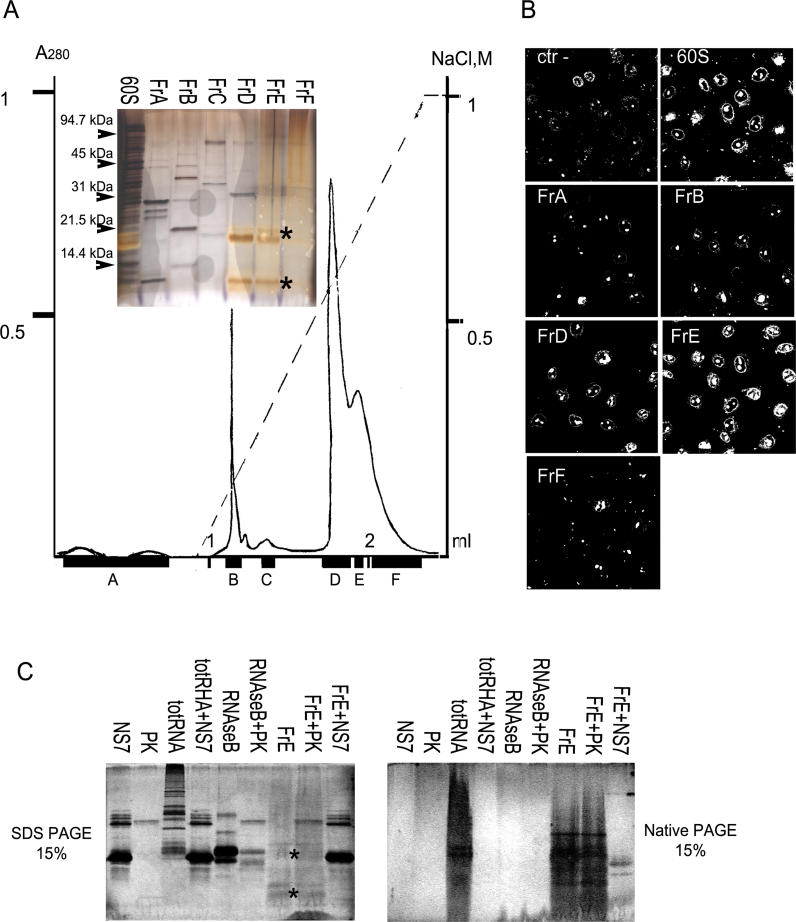
Purification and Characterization of the Active Component in the 60S (A) Chromatographic profile obtained after hydrophobic interaction and ion exchange chromatography of the original 60S (see Materials and Methods). Individual fractions were analyzed by SDS-PAGE and silver staining (60S, 20-μg protein/lane, and Fractions (Fr) A to F, 10-μg protein/lane). Asterisks indicate the two bands present in FrE. (B) Nuclear import of YOYO-1–labelled RTCs in permeabilized HeLa cells in the presence of 1× energy-regenerating system, 60S, fractions FrA to FrF (0.5 mg/ml) or buffer (ctr –). (C) The two bands in FrE are nucleic acids. Equal amounts of FrE were subjected to proteinase K (PK) or nuclease S7 (NS7) treatment (see Materials and Methods) and analyzed by SDS-PAGE or native PAGE followed by silver staining. Total HeLa RNA (totRNA) and RNAse B were used as controls for nuclease or proteinase digestions, respectively.

The 60S fraction was further fractionated by chromatography. 60S was bound to a hydrophobic interaction column (HIC), eluted by a 60% to 0% AS gradient, dialysed extensively, and tested in the nuclear import assay. The fraction with RTC import ability eluted from the column at approximately 50% AS (unpublished data). The active fraction from HIC was then loaded onto an ion exchange column and eluted with a 0 to 1M NaCl gradient. Six main fractions were obtained and analyzed by SDS-PAGE and silver stain, and by the nuclear import assay (Figure 2A and 2B). As shown in Figure 2B, fraction E, which eluted from the column with 0.5 M NaCl, was the most active in inducing RTC nuclear import in permeabilized HeLa cells. Fraction D also had activity, but lower than fraction E. After silver stain of the protein gel, it became apparent that fraction E contained two main bands migrating at approximately 20 and 12 KDa (Figure 2A). Surprisingly, the two bands had an unusual brownish colour instead of the black typical of proteins visualized by silver stain. The brownish colour is seen with glycoproteins or lipoproteins, but all our attempts to detect glycans or lipid residues were unsuccessful. Upon closer inspection of the gel, we observed that fraction F contained a smear of the same colour, highly suggestive of nucleic acids, hence fraction E was digested with proteinase K or nuclease S7, and then analyzed by SDS or native PAGE. As shown in Figure 2C, proteinase K digested a control protein (RNAseB), but had no effect at all on fraction E. On the other hand, nuclease S7 digested almost completely fraction E as well as the total RNA control. Thus fraction E contained nucleic acids.

### RNA Is the Active Component in the 60S

**Figure 3:**
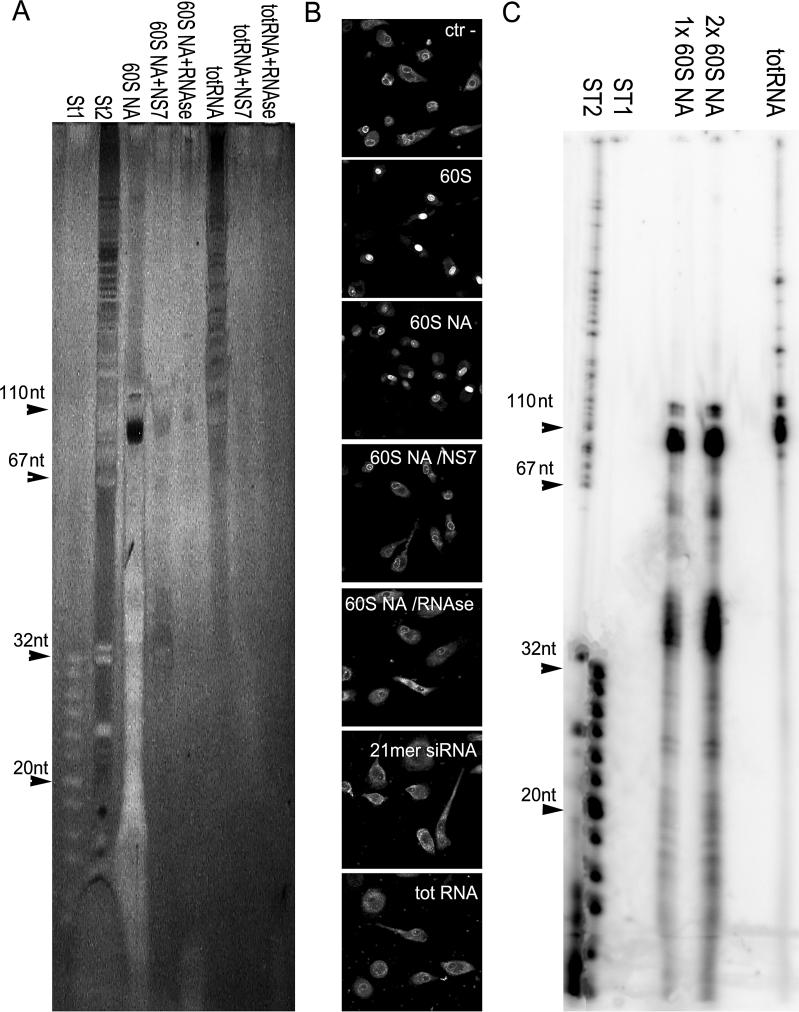
The 60S NA Fraction Contains Small RNA Molecules (A) Equal amounts of nucleic acids purified from the active Phenyl-Sepharose fraction (60S NA) were subjected to nuclease S7 (NS7) or DNAse-free RNAse (RNAse) treatment and analyzed by 15% denaturing PAGE followed by silver staining. Total HeLa RNA (totRNA) was used as control for nuclease and RNAse treatments. ST1, oligonucleotide size markers (range, 8–32 nucleotides); ST2, size markers pBP322DNA-MspI. (B) 60S NA loses its ability stimulate RTC nuclear import after nuclease or RNAse treatment. Nuclear import of YOYO-1 labelled RTCs in permeabilized primary human macrophages in the presence of 1× energy-regenerating system and 60S (0.5 mg/ml), 60S NA (1 μg), 60S NA digested with NS7, 60S NA digested with RNAse (1-μg starting material), 21mer siRNA (1 μg), total HeLa RNA (totRNA, 1μg), or buffer (ctr –). (C) 60S NA fraction can be specifically 3′-end radiolabelled by T4 RNA ligase. Following 3′-end labelling with 5′-[^32^P]pCp, samples were analyzed by 15% denaturing PAGE and visualized by Storm 860 PhosphoImager. Total HeLa RNA (totRNA) was used as a control for T4 RNA ligase reaction. ST1 and ST2 are 5′-end radiolabelled oligonucleotide size markers as in (A).

To find out if nucleic acids were DNA or RNA, the active fraction (this time obtained by 60% AS precipitation and HIC chromatography) was digested by proteinase K and extracted by phenol/chloroform. The resulting sample (hereafter called 60S nucleic acids, 60S NA) was first treated with nuclease S7 or with DNAse-free RNAse, and the nucleic acids re-purified by treatment with proteinase K followed by phenol/chloroform extraction and ethanol precipitation. Samples were then analyzed by SDS-PAGE and silver stain. As shown in Figure 3A, the 60S NA fraction contained a main band migrating with an apparent size of approximately 80 nt, in addition to smaller bands migrating around 40 to less than 20 nt. Both nuclease S7 and DNAse-free RNAse digested the 60S NA, suggesting that RNA was its component (Figure 3A). The 60S fraction and the 60S NA treated with nucleases and re-purified as described earlier were also tested in the nuclear import assay. As shown in Figure 3B, 60S induced strong RTC nuclear import in permeabilized primary human macrophages, the 60S NA fraction was also clearly active, albeit at reduced levels, whereas the nuclease-treated 60S NA fraction was inactive, similar to a control 21mer siRNA or total RNA. Moreover, the 60S NA prepared from Jurkat and SupT1 lymphocytic cells was also active in inducing RTC nuclear import, indicating that this activity was not limited to HeLa extracts (Figure S1). We further confirmed that the 60S NA contained small RNA molecules by 3′-end radiolabelling of the samples with T4 RNA ligase and analysis by denaturing PAGE. Figure 3C shows the resulting radiolabelled bands ranging in size from approximately 100 to less than 20 nt.

### The 60S NA Fraction Contains tRNA-Like Molecules

**Figure 4:**
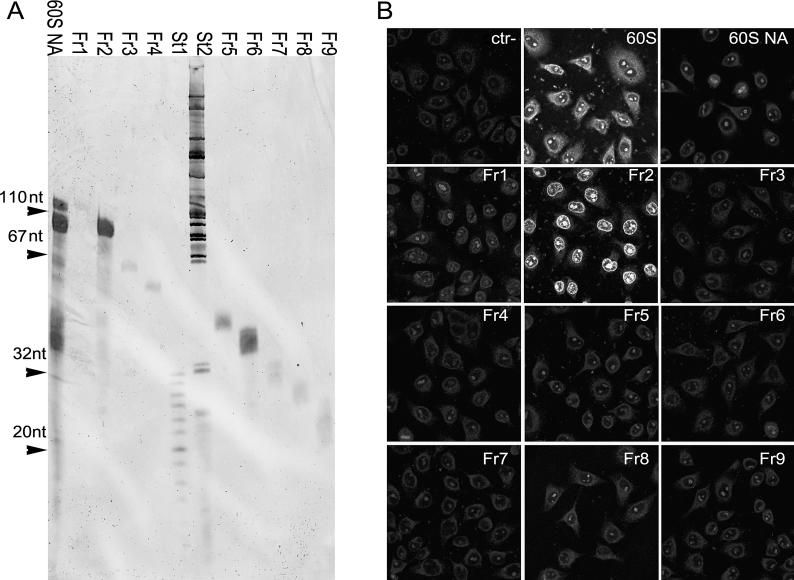
Size Fractionation of the Small RNA Molecules in the 60S NA (A) The 60S NA was separated onto a 15% denaturing gel, and individual bands were cut, eluted from the gel, and re-loaded onto a 15% denaturing gel to confirm correct size fractionation. Bands were labelled by SYBR gold staining and visualized by Storm 860 PhosphoImager. ST1 and ST2, oligonucleotide size markers, as in Figure 3A. (B) Nuclear import YOYO-1–labelled RTCs in permeabilized HeLa cells in the presence of 1× energy-regenerating system and 60S (0.5 mg/ml), 60S NA (100 ng), the nine main bands eluted from the gel as shown in (A) (140 nM each), or buffer (ctr–).

Figure 3C shows that the 60S NA contained several small RNAs, and we therefore attempted to isolate and clone the active species. The 60S NA fraction was size-fractionated by denaturing PAGE and visualized by SYBR gold staining. Each band was then cut, eluted from the gel and re-examined by denaturing PAGE to confirm its size and by the nuclear import assay to confirm its activity. As shown in Figure 4A, the nine main bands present in the 60S NA were correctly fractionated and eluted from the gel. The concentration of all samples was equalized prior to the nuclear import assay. Importantly, fraction 2, migrating with an approximate size of 80 nt, showed the highest RTC nuclear import activity (Figure 4B). Having isolated the most active fraction, we adapted a method described by Elbashir et al. [22] to clone it. Briefly, an adaptor was ligated to the 3′ end of fraction 2, and the sample was gel purified. All our attempts to ligate a 5′-end adaptor were unsuccessful, so the sample with the 3′-end adaptor was used as a template for first strand cDNA synthesis, and this was followed by addition of a polyC tail to its 3′ end by terminal transferase. The template was then amplified by PCR (using proof-reading *Taq* polymerase), cloned, and sequenced.

Of more than 100 sequences examined, 31 distinct clones were obtained, and several of them were severely or partially truncated, presumably due to RT pausing at hypermodified nucleotides (Table 1). A sequence alignment of the 31 clones indicated that they all belonged to the same family of small RNAs, and a BLAST search of the human genome database indicated that they were up to 97% homologous to some human tRNAs (Table 1). In particular, of 31 clones, 11 were highly homologous to tRNA^Gly^, six to tRNA^Lys1,2^, four to tRNA^Glu^, three to tRNA^Val^, and two to tRNA^Ala^, tRNA^Tyr^, and tRNA^Arg^. We could not find perfectly matching sequences in the human genome database, suggesting that these small RNAs did not represent a new class of tRNAs genes, and that small differences were most likely due to artefacts during reverse transcription [23]. Remarkably, all clones except two (E1 and H1) lacked the complete 3′ CCA end typical of mature tRNAs. Moreover, compared to typical tRNAs, most clones had the 5′ end shortened by one or more nucleotides, with the exception of clone B3 that had a longer than normal 5′ end (Table 1). Presumably this is due to RT pausing during first strand cDNA synthesis. The tRNAscan-SE search server (Washington University) predicted all our clones to fold into the typical tRNA cloverleaf secondary structure, with the exception of the truncated ones (unpublished data) [24].

**Table 1:**
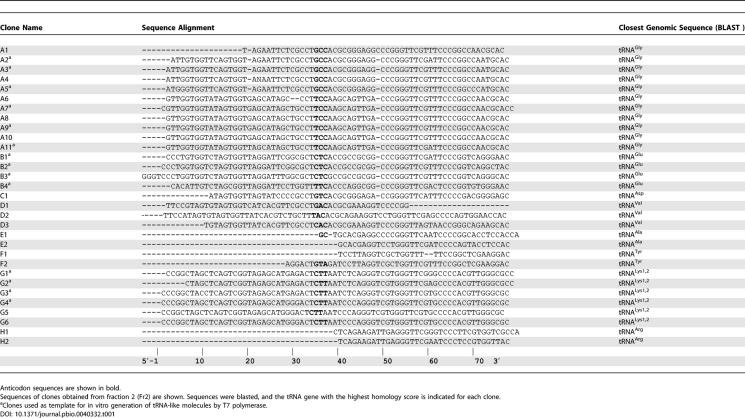
Sequence of Clones from Fraction 2

**Figure 5:**
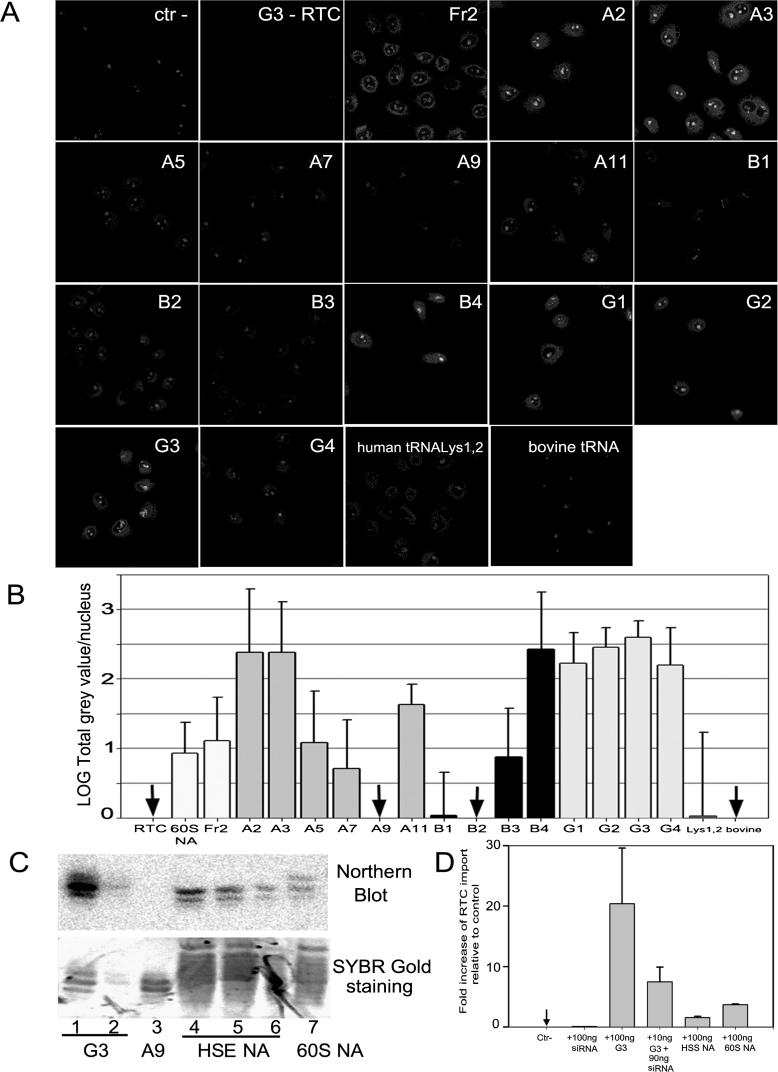
Nuclear Import Activity of Small RNA Molecules Generated In Vitro (A) Nuclear import of YOYO-1–labelled RTCs in permeabilized HeLa cells in the presence of 1× energy-regenerating system and Fr2 (100 ng), the indicated small RNAs generated by in vitro T7 transcription (see Table 1, Protocol S1, and Table S2) (140 nM each, corresponding to ~100 ng), human tRNA^Lys1,2^ (100 ng), bovine tRNAs (100 ng), or buffer (ctr–). Nuclear import of the G3 RNA molecule in the absence of YOYO-1–labelled RTCs (G3 − RTC) was used as an additional negative control. (B) Quantification of RTC nuclear import as shown in (A). Images acquired by confocal microscopy were analyzed by MetaMorph software version 4.5r4 (Universal Imaging Corp) and the total fluorescence of the nuclei divided by the number of cells per field (see also Figure S2). At least 150 cells were counted per experiment. Bars represent the log mean fluorescence per nucleus ± standard deviation of six independent experiments. (C) Quantification of G2/G3 RNA in HSE NA and 60S NA by Northern blot. RNA was separated by 15% denaturing PAGE, transferred onto a nylon membrane, and probed with an oligonucleotide complementary to G2/G3 RNA. There were 100-ng G3 RNA (lane 1), 10-ng G3 RNA (lane 2), 100-ng negative control A9 RNA (tRNA^Gly^) (lane 3), 700-ng HSE NA (lane 4), 350-ng HSE NA (lane 5), 175-ng HSE NA (lane 6), and 200-ng 60S NA (lane 7). Upper panel, hybridized membrane visualized by Phosphoimager, lower panel, SYBR Gold staining of denaturing PAGE. Image Quant (Molecular Dynamics) was used to calculate the intensity of the signals. (D) Comparative RTC nuclear import activity of HSE NA (100 ng), 60S NA (100 ng), G3 RNA (100 ng), G3 RNA (10 ng + 90-ng carrier siRNA), buffer (ctr–), or buffer + 100-ng carrier RNA. Nuclear import assays were performed in permeabilized HeLa cells in the presence of labelled RTCs, 1× energy mix, and the indicated RNAs. Bars represent the fold nuclear import increase above background (RTC + energy mix) ± standard deviation of two independent experiments.

To investigate which of these RNA molecules were able to support nuclear import of HIV-1 RTCs, we have selected 14 sequences from “full length” clones belonging to the three most abundant species, i.e., with homology to tRNA^Gly^, tRNA^Glu^ and tRNA^Lys1,2^ and transcribed them in vitro from a T7 promoter (Table 1). Since T7 polymerase can add extra non-template nucleotides to the 3′ ends of transcripts, all T7 products were carefully purified in long denaturing gels using standards of known size, eluted, ethanol-precipitated, and used in the nuclear import assay to test their ability to induce RTC nuclear import. Of 14 RNAs analyzed, seven showed good import activity (A2, A3, B4, and G1–G4), four had intermediate activity, and three had weak activity (A9, B1, and B2) (Figure 5A). To better quantify import activity of the various RNA molecules, confocal images from six independent nuclear import experiments were analyzed by MetaMorph to calculate the mean fluorescent value per cell nucleus, an indirect measure of the efficiency of RTC nuclear import (see also Figure S2). As shown in Figure 5B, all the RNA molecules with homology to tRNA^Lys1,2^ were very active in inducing RTC nuclear import. However, only two RNAs with homology to tRNA^Gly^ and one with homology to tRNA^Glu^ showed a similar degree of activity. The RTC nuclear import activity of the G3 RNA was confirmed by fluorescent in situ hybridization (FISH) with a probe spanning the HIV-1 vector genome (Figure S3). The G3 RNA was active in inducing HIV-1 RTC, but not MLV RTC nuclear accumulation (Figure S4). Interestingly, wild-type tRNA^Lys1,2^ had low nuclear import activity (the high standard deviation for tRNA^Lys1,2^ in Figure 5B is due to nuclear import activity above background in one out of six experiments). This could also be due to rapid nuclear import and re-export of such tRNAs resulting in a “neutral” phenotype in our experimental conditions. The Ran mix was not required for tRNA-mediated nuclear import of RTCs (unpublished data).

To examine the specific activity of the RNA molecules transcribed in vitro, we estimated the amount of G2/G3 RNAs in the 60S NA fraction and in the HSE nucleic acids (NA) preparation by Northern blot and Phosphoimager analysis (Figure 5C) (sequence similarity prevented us from distinguishing between G2 and G3 RNAs). A9 tRNA (tRNA^Gly^) was used as control for specific hybridization. The G2/G3 RNAs represented approximately 10% of total small RNA loaded on the gel or 10 ng per 100 ng of 60S NA and HSE NA (these fractions contained different RNA species so no accurate value of their molarity could be obtained). Quantitative analysis of RTC nuclear import indicated that 10ng of G3 RNA (corresponding to 14nM) had a similar or higher activity than 100ng of 60S NA and HSE NA (Figure 5D). Moreover, the amount of HSE sufficient to support RTC nuclear accumulation in permeabilized cells contained 80–100 ng small RNA (unpublished data). Thus it appeared that synthetic and endogenous tRNAs were similarly active. Synthetic tRNAs lacked hypermodification, hence these results suggested that tRNA post-transcriptional modification may not be critical for nuclear import activity.

To investigate the specificity of tRNA-mediated RTC nuclear import, the M9 peptide fused to the maltose binding protein (MBP) was chosen as an alternative substrate. M9 is a nuclear import signal for hnRNP A1 recognised by transportin, and it was chosen because its mechanism of nuclear import is mainly Ran-independent [25–27]. MBP-M9 nuclear import was supported by transportin, but not by 60S NA, Fraction 2, and G3 and B2 RNAs added at a concentration sufficient for maximal activity with RTC (Figure S5). Thus, these RNA molecules were specifically inducing RTC nuclear import.

### The T- and Anticodon Arms Are Required for Nuclear Import Activity

**Figure 6:**
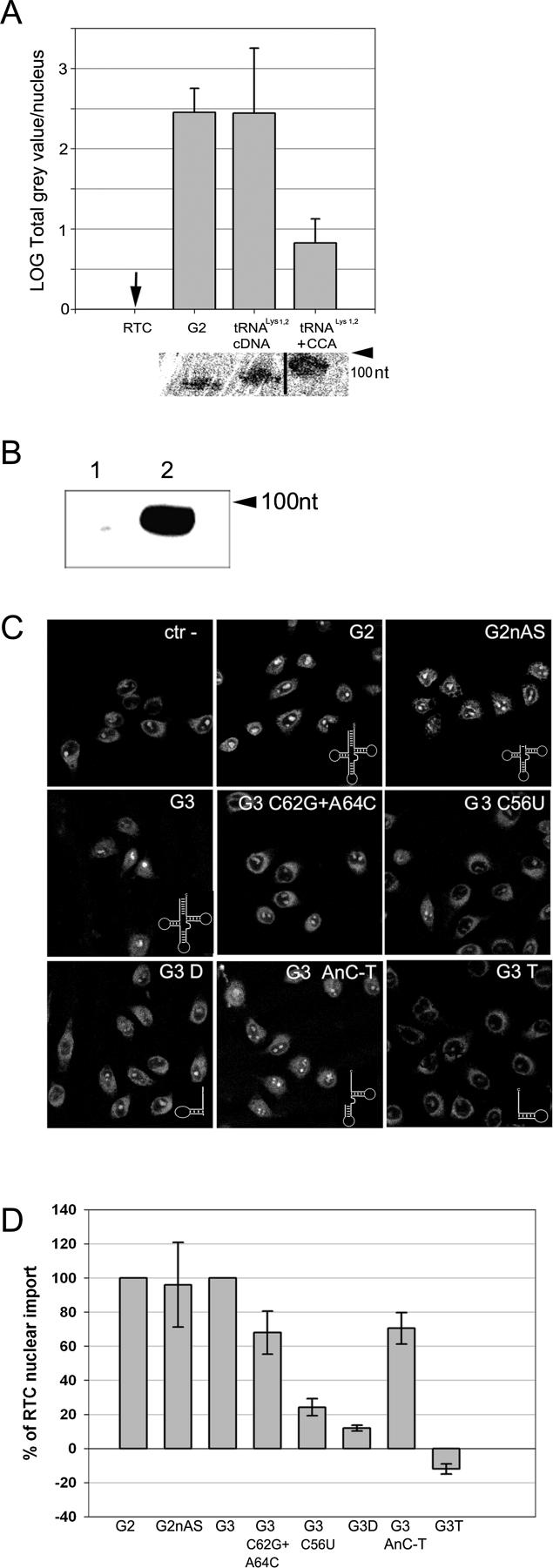
Structural Requirements of tRNAs for RTC Nuclear Import Activity (A) Quantification of RTC nuclear import in the presence of G2 RNA, modified G2 RNA, and modified G2 RNA with 3′ CCA end. Quantification was performed as in (D) and bars represent the mean log fluorescence value of the nuclei ± standard deviation of five independent experiments. Correct generation of the tRNA^Lys1,2^-size G2 RNA and addition of the CCA tail to this RNA was monitored by long 15% denaturing PAGE followed by SYBR Gold staining (bottom panel). (B) The presence of the correct 3′ CCA tail in the tRNA ^Lys1,2^ + CCA molecule was monitored by splint labelling. The tRNA ^Lys1,2^ + CCA RNA was purified by denaturing PAGE as shown in (A), bottom. Following splint labelling with [α-^32^P]dATP, the G2 + CCA RNA was re-analyzed on a 15% denaturing PAGE and visualized by PhosphoImager (see Protocol S1). Labelling was performed with antisense primer −CCA (lane 1), and with antisense primer +CCA (lane 2). (C) Nuclear import of YOYO-1–labelled RTC in permeabilized HeLa cells in the presence of 1× energy-regenerating system and mutant RNAs generated by in vitro T7 transcription (140 nM each) (see Tables S1 and S2) or buffer (ctr–). G2, original RNA; G2nAS, mutant with shortened acceptor stem; G3, original RNA; G3 C56U, point mutation disrupting D- and T-loops pairing; G3 C62G + A64C, double point mutation disrupting T-stem secondary structure; G3 AnC-T, deletion mutant lacking the D-arm; G3D, deletion mutant lacking anticodon loop and T-arm; G3T, deletion mutant lacking D- and anticodon arms. (D) Quantification of RTC nuclear import with the same RNA mutants as shown in (C). Images were acquired by confocal microscopy and analyzed using the MetaMorph software version 4.5r4 (Universal Imaging Corp) and the total fluorescence in the nucleus divided by the number of cells per field. At least 150 cells were counted per experiment. Bars represent the percentage of total fluorescence per nucleus relative to control (G2 and G3 = 100) ± standard deviation of three independent experiments.

It was surprising to find that wild-type tRNA^Lys1,2^ appeared to have little RTC nuclear import activity compared to our very homologous RNA molecules, and we wondered if the CCA end had any effect on this phenotype. A variant of the G2 clone was generated by PCR to make it identical to the wild-type tRNA^Lys1,2^ except for the 3′ CCA end, then a CCA tail was specifically added to its 3′ end. These mutant RNAs were synthesized in vitro by T7 transcription and purified in denaturing gels (see the supplementary materials and methods [Protocol S1] and Tables S1 and S2). The addition of the CCA tail was confirmed by assessing size mobility in long 15% denaturing PAGE against standards of known size (Figure 6A, bottom panel). The presence of a correct CCA tail in the artificially synthesized tRNA^Lys1,2^ size +CCA G2 RNA variant was confirmed by 3′-end splint labelling with two oligonucleotides complementary to the 3′ end of G2 RNA with or without the CCA tail, respectively [28,29] (Figure 6B and Protocol S1). Taken together these results indicated that the bulk of the gel-purified RNAs contained the correct nucleotide additions. When tested in nuclear import assays, the original and the variant G2 RNAs lacking the 3′ CCA end had identical RTC import activity; however, addition of the 3′ CCA end to the G2 RNA variant resulted in reduced RTC import activity (Figure 6A).

To gain a better understanding of the structure/function relationship contributing to the RTC nuclear import activity of these tRNAs, several deletion and point mutants were generated using the G2 and G3 clones as template (see Protocol S1 and Tables S1 and S2). Mutant clones were generated by PCR, confirmed by sequencing, transcribed in vitro by T7 RNA polymerase, purified as before, and examined in nuclear import assay experiments. Shortening the acceptor stem had no significant effect on the G2 RNA molecule ability to induce RTC nuclear import (Figure 6C, panel G2nAS [upper right], and Figure 6D). A deletion mutant lacking the D-arm (G3 AnC-T) lost some activity, a deletion mutant containing the D-arm only (G3 D) was almost completely inactive, and another deletion mutant containing the T-arm only (G3 T) (minus CCA end) had a dominant negative activity (Figure 6C, panels G3 AnC-T, G3 D, and G3 T [bottom row], and Figure 6D). The dose-dependent dominant negative effect of the G3 T mutant was confirmed in competition assays where more than 70% inhibition of RTC import was achieved at a 1:1.3 ratio of G3 and mutant G3 T RNAs (Figure S5). Addition of the full 3′ CCA end to the G3 T mutant resulted in partial loss of the dominant negative phenotype (Figure S6). The G3 T RNA molecule could not inhibit M9 nuclear import in the presence of transportin at the same dose used to completely inhibit RTC nuclear import, demonstrating substrate specificity (Figure S5). Furthermore, disruption of the T-stem secondary structure by point mutations (G3 C62G+A64C) reduced RTC nuclear import activity and disruption of the correct T-loop/D-loop pairing (G3 C56U) had a more severe inhibitory effect (Figure 6C and 6D). These tRNA point mutants have been previously demonstrated to be as stable as wild-type tRNA [30–32]. Overall these results suggested that correct tRNA folding is important for RTC nuclear import activity together with the anticodon loop and the T-arm. The acceptor stem appeared not to be required, and the 3′ CCA end appeared to be inhibitory in the conditions tested.

### Defective tRNAs Accumulate into the Nucleus on Their Own in an Energy- and Temperature-Dependent Way

**Figure 7:**
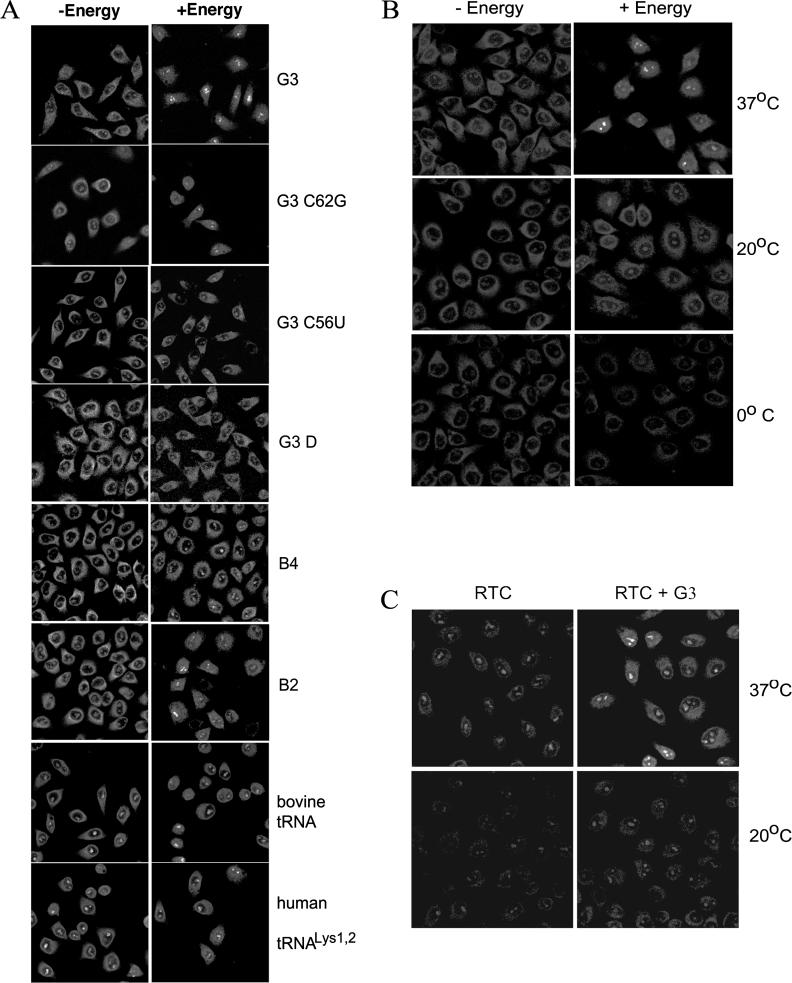
In Vitro Generated tRNAs Accumulate into the Nucleus of Permeabilized HeLa Cells in an Energy- and Temperature-Dependent Way (A) Nuclear import assay in permeabilized HeLa cells in the presence or absence of 1× energy-regenerating system and YOYO-1–labelled RNA molecules and their mutants (see Tables S1 and S2 and Figure 6) (140 nM each, corresponding to 100 ng), human tRNA^Lys1,2^ (100 ng), and bovine tRNAs (100 ng). Nuclear import assay of the G3 D mutant RNA was performed in the presence of 100 ng inactive carrier RNA (21mer RNA). (B) Nuclear import assay in the presence of 140 nM YOYO-1–labelled G3 RNA performed at different temperatures with or without an energy-regenerating system. (C) Nuclear import assay with labelled RTCs and buffer (RTC) or 140 nM G3 RNA (RTC + G3) performed at different temperatures in the presence of an energy-regenerating system.

We then investigated if these tRNAs species might induce RTC nuclear import by functioning like adaptors. In vitro synthesized tRNA molecules were directly labelled with the nucleic acids dye YOYO-1 after gel purification, dialysed, and tested in the nuclear import assay in the absence of RTCs. Native PAGE showed that the various tRNAs incorporated YOYO-1 equally well (unpublished data). As shown in Figure 7A, the G3 RNA efficiently accumulated into the nucleus only in the presence of an energy-regenerating system. The G3 C62G point mutant tRNA accumulated less efficiently than G3 tRNA into the nuclei, and the G3 C56U point mutant tRNA showed almost background levels of nuclear signal, consistent with data showed in Figure 6D. Wild-type human tRNA^Lys1,2^ and bovine tRNAs were not seen to accumulate appreciably into the nuclei in an energy-dependent way, consistent with previous reports [33]. Interestingly, one of the tRNA species that had very low RTC import activity could still efficiently accumulate into the nuclei on its own (compare Figure 5B and Figure 7A, B2 RNA), suggesting a specific interaction defect with the RTC. The G3 D RNA mutant remained in the cytoplasm (Figure 7A). These results further suggested that the T-arm plays an important role in nuclear accumulation of the tRNAs and that it is likely to be required for interaction with one or more cellular factors, which may be part of a nuclear import pathway. They also showed that nuclear accumulation of these tRNA species is strictly energy dependent. Moreover, G3 RNA efficiently accumulated into the nuclei only if nuclear import assays were carried out at 37 °C. If assays were carried out at 20 °C, a lower nuclear signal was observed, and G3 RNA mainly accumulated at the nuclear periphery. At 0 °C G3 RNA was not found in the nuclei at all (Figure 7B). Labelled RTCs followed a similar pattern in the presence of G3 tRNA (Figure 7C). The fact that nuclear accumulation of these tRNA species was strictly energy and temperature dependent supports the surprising possibility that they might be actively imported and did not accumulate in the nuclei simply by diffusion and selective nuclear retention [34].

### HIV-1 Ability to Infect Non-Dividing Cells and Incorporation of Active tRNA Species May Depend on *gag*

**Figure 8:**
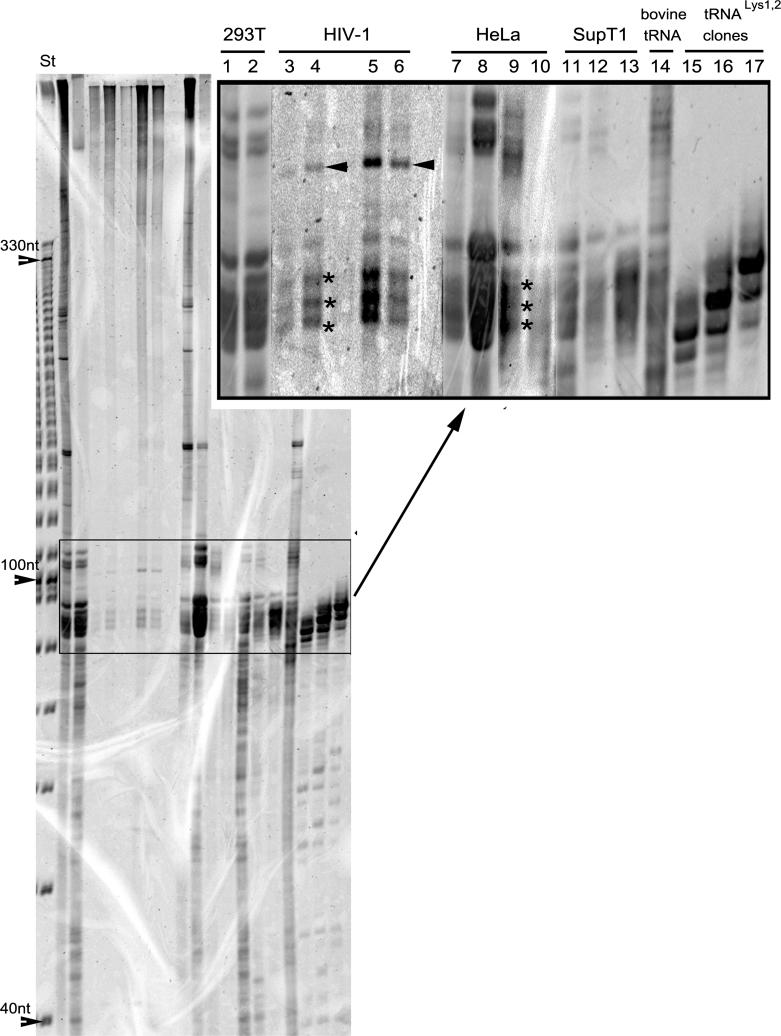
Profile of Small RNA Species Incorporated into HIV-1 Particles Total and small RNA were obtained from cells and purified virions, and analyzed onto a long (50 cm) 15% denaturing PAGE followed by SYBR Gold staining. There was a 10-bp DNA ladder (lane St), 2.3-μg total 293T RNA (lane 1), 1.5-μg small 293T RNA (lane 2), purified HIV-1 virion RNA (169 ng and 338 ng) (lanes 3 and 4, respectively), purified RNA from HIV-1 virions not containing the viral genome (472 ng and 236 ng) (lanes 5 and 6, respectively), 2.7-μg total HeLa RNA (lane 7), 2.4-μg small HeLa RNA (lane 8), 100-ng 60S NA fraction from HeLa cells (lane 9), 100-ng Fr2 from HeLa cells (lane 10), 0.9-μg SupT1 small RNA (lane 11), 200-ng 60S NA fraction from SupT1 cells (lane 12), 200-ng Fr2 from SupT1 cells (lane 13), 1.5-μg bovine tRNA (lane 14), 200-ng tRNA^Lys1,2^ size G2 RNA + C tail (see Table S2) (lane 15), 200-ng tRNA^Lys1,2^ size G2 RNA + CC tail (lane 16), and 200-ng tRNA^Lys1,2^ size G2 RNA + CCA tail (lane 17). Asterisks indicate positions of similar size bands in different lanes. Arrows indicate small RNAs specifically enriched in purified virions. The contrast in lanes 3 to 6 in the magnified rectangle has been artificially increased to help in visualizing bands.

All retrovirus particles contain small RNA molecules, most of which are tRNAs of cellular origin (reviewed in [35]). Thus we investigated if the tRNAs with RTC nuclear import activity were also incorporated into HIV-1 particles. Large amounts of high-titer virus stocks were generated by transfecting 293T cells and were purified by two rounds of density sedimentation in sucrose gradients (see Protocol S1). Analysis by electron microscopy confirmed the purity of the resulting samples (unpublished data). Nucleic acids were extracted from purified viral samples and analyzed for the presence of small RNAs. Repeated attempts to distinguish mature cellular tRNAs from tRNA having a C or CC 3′ end by 3′-end splint labelling [29] were unsuccessful, and the high homology of the two species prevented us from using differential hybridization. Thus, we took advantage of the fact that some of the tRNAs lacked the complete 3′ CCA end, and we ran samples onto sequencing gels (Figure 8).

Comparison of the total and small cellular RNA species with that extracted from purified HIV-1 virions revealed that viral-specific RNAs were enriched in our samples and that some cellular RNAs were excluded. For example, a band migrating at approximately 120 nt was clearly visible in viral samples. but not or barely visible in cellular samples (from 293T cells), even if 20-fold more cellular RNA was loaded on the gel (Figure 8, compare lanes 1 and 2 with lanes 3 to 6). Importantly, viral samples contained three distinct bands migrating at the level of cellular tRNAs. These three bands were present also in the 60S NA and in fraction 2 and migrated at a similar level of an artificially synthesized tRNA^Lys1,2^ having a single 3′ C end, or a CC or a full CCA end, respectively (Figure 8, compare lanes 3 to 6 to lane 9 to lanes 15–17). Incorporation of these small RNA species occurred independently of the viral genome since they were found also in virions made in the absence of the vector genome itself (Figure 8, lanes 5 and 6). The presence of G3 RNA in the lower migrating band (+C) of the viral preparations was confirmed by Northern blot (unpublished data).

**Figure 9:**
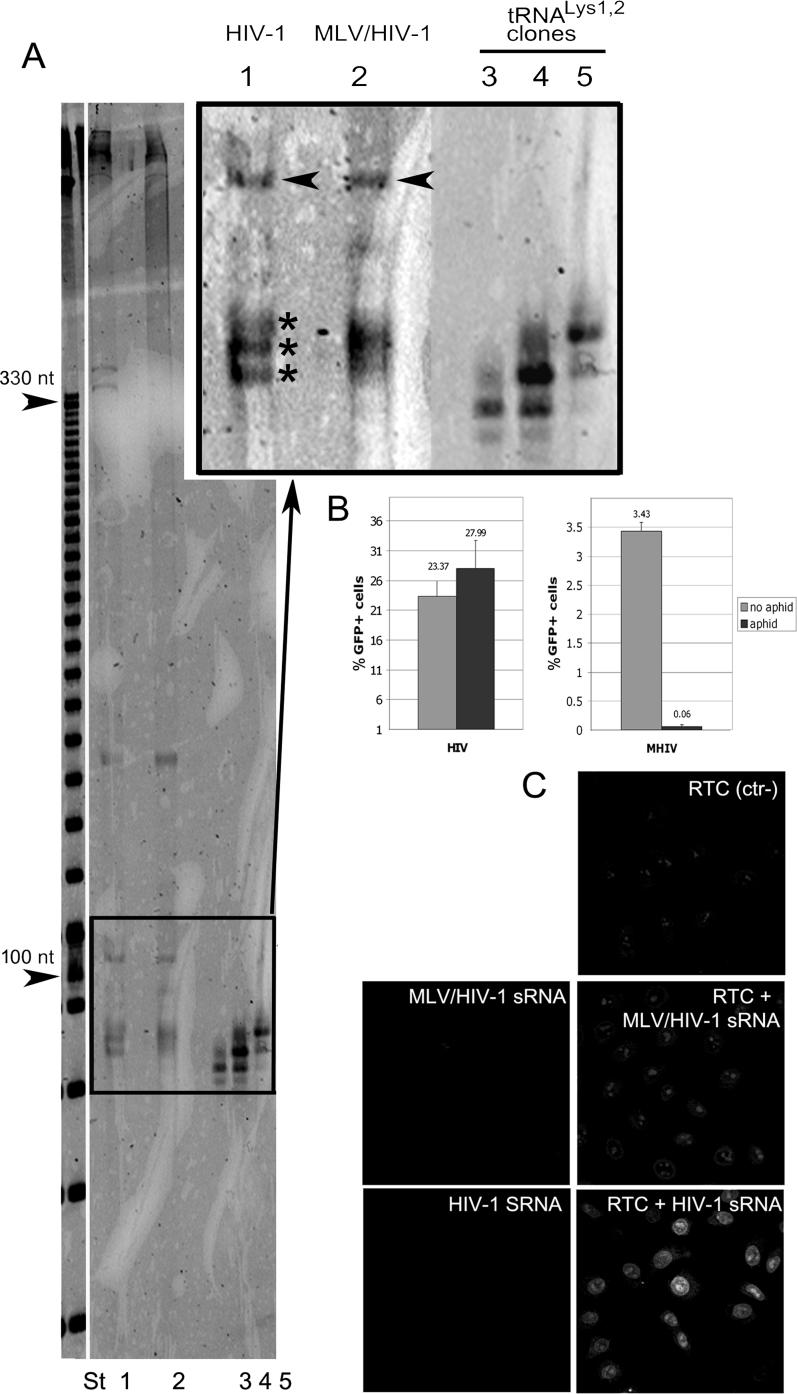
MHIV-1 *gag* Mutant Does Not Incorporate tRNA Species with RTC Nuclear Import Activity (A) Viral RNA (1.5 μg) was extracted from purified HIV-1 (lane 1) or MHIV-1 *gag* mutant (lane 2) and separated onto a long (50 cm) 15 % denaturing PAGE followed by SYBR Gold staining. There was a 10-bp DNA ladder (lane St), 25-ng tRNA^Lys1,2^ size G2 RNA + C tail (see Table S2) (lane 3), 25-ng tRNA^Lys1,2^ size G2 RNA + CC tail (lane 4), and 25-ng tRNA^Lys1,2^ size G2 RNA + CCA tail (lane 5). Asterisks indicate the three small RNA bands found in HIV-1; arrows indicate viral-specific small RNA molecule common to both viruses. (B) Single-cycle infection assays in cell cycle–arrested cells. Cells were treated with aphidicolin for 24 h to induce G1/S arrest, infected with the same dose of wild-type HIV-1 or MHIV *gag* mutant, and analyzed for GFP expression by flow cytometry 24 h after infection. Bars represent the average value ± standard deviation of two experiments. (C) Nuclear import of YOYO-1–labelled HIV-1 RTCs in permeabilized HeLa cells in the presence of 1× energy-regenerating system and buffer (ctr–) or HIV-1 small RNAs (HIV-1 sRNA) or MHIV-1 *gag* mutant small RNAs (MLV/HIV-1 sRNA) (30ng + 70-ng carrier siRNA) after elution from the gel. Nuclear import of the eluted small viral RNAs from wild-type HIV-1 and MHIV *gag* mutant in the absence RTCs was performed as an additional negative control (HIV-1 sRNA and MLV/HIV sRNA, respectively).

Recently, the capsid protein (p24 CA) has been shown to be a crucial determinant for HIV-1 nuclear import, and HIV-1 mutants in which the complete *gag* region was substituted by MLV *gag* were unable to infect non-dividing cells [13]. Thus, we investigated if such a HIV-1 *gag* mutant incorporated tRNA species with RTC nuclear import activity. Wild-type HIV-1 and the MHIV-mMA12CA mutant containing MLV *gag* [13] were concentrated, purified, and used to infect cells arrested in the cell cycle by treatment with aphidicolin. In agreement with a previous study [13], cell cycle arrest dramatically inhibited MHIV-mMA12CA virus infection efficiency, but had no effect on HIV-1 infection efficiency. RNA was extracted from purified virus as described earlier and analyzed by long denaturing PAGE. HIV-1 viruses contained some tRNA species that migrated differently from tRNAs present in the mutant MHIV *gag* virus (Figure 9). tRNAs were excised from the gel, eluted, and tested in the nuclear import assay. As shown in Figure 9B, tRNAs eluted from wild-type HIV-1 induced significant RTC nuclear import, but tRNAs eluted from the MHIV-1 *gag* mutant were almost completely inactive. These results suggested that the MHIV-1 *gag* mutant virus might not be able to incorporate and/or interact with tRNA species competent for RTC nuclear import. Alternatively, tRNAs obtained from the MHIV-1 *gag* mutant virus may be unable to interact with HIV-1 RTCs and conversely the mutant RTCs might not have the right structure to be actively imported into the nucleus, despite the presence/incorporation of tRNAs competent for nuclear import.

## Discussion

We have found that some tRNA species promoted nuclear import of HIV-1 RTCs. Several lines of evidence support these conclusions. First, small RNAs isolated by chromatography from cytosolic extracts induced nuclear import of RTCs, and RNAse treatment of the samples eliminated import activity. Second, only one species of small RNAs had nuclear import activity following size fractionation of the active sample in denaturing gels, thus excluding a non-specific effect due to the presence of small RNA molecules in the import assay. Third, the same RNA molecules synthesized in vitro were active, and import activity could be mapped to the anticodon and T-arm regions. Importantly, the structural requirements were the same for nuclear import of RTCs and nuclear accumulation of the tRNAs on their own, making it unlikely that tRNAs were simply titrating out an inhibitor of RTC nuclear import. Lastly, tRNA species with HIV-1 RTC nuclear import activity were incorporated into and recovered from HIV-1 viral particles.

It is not completely clear at present how these tRNAs species may promote nuclear import of HIV-1 RTCs; nonetheless, some insight may be gained from the available data. The in vitro synthesized tRNAs efficiently accumulated into the nuclei of permeabilized cells in a strictly energy- and temperature-dependent way. A very similar temperature dependence was observed for tRNA nuclear export and is consistent with a carrier-mediated translocation mechanism [36]. Moreover, these tRNA species promoted nuclear import of the RTC, a complex too large to diffuse through nuclear pore complexes. Hence it is unlikely that tRNA accumulate in the nuclei by passive diffusion and selective nuclear retention. Overall, our results support the surprising possibility that at least some tRNA species may be actively uptaken into the nucleus of human cells. We note that both normal and CCA-less tRNAs have recently been reported to actively shuttle between nucleus and cytoplasm in yeast cells [37,38]. In our experimental conditions, RTC and tRNA nuclear accumulation was also reliant on structural features because point mutant tRNA molecules, in which the correct T-stem folding was disrupted, were found in the nuclei at reduced levels and had low RTC import activity. Furthermore, a minihelix made of the T-arm only had dominant negative activity. It is conceivable that the properly folded T-arm interacts with putative cellular factors important for nuclear import of the tRNAs whereas the anticodon arm may be more important for interaction with the RTC. This is supported by the fact that the highly homologous B1, B2, and B3 tRNAs were inactive in inducing RTC nuclear import, but B2 tRNA could still be efficiently imported into the nucleus on its own, suggesting a defect in RTC binding. On the other hand, B4 tRNA was clearly active in stimulating RTC nuclear import and differed from B1, B2, and B3 tRNAs in the anticodon loop. However, the anticodon itself is not sufficient to confer specific RTC binding since at least some tRNA^Gly^, tRNA^Glu^, and tRNA^Lys1,2^ had RTC nuclear import ability. The very low nuclear import of MLV RTCs induced by G3 tRNA also suggests that specific interactions are required between some tRNAs and their RTC substrate.

In light of these observations, the simplest model to explain tRNA-mediated nuclear import of RTCs is based on multiple interactions between the anticodon arm and the viral complex on one hand and the T-arm and putative cellular factors promoting nuclear import on the other hand. Interestingly, the T-arm is also important in tRNA nuclear export since the same mutations in the T-stem-loop that we have tested also abolish interaction with exportin-t [31,32]. Moreover, the fact that the T-stem secondary structure requirements for RTC and tRNAs nuclear accumulation were the same argue against the possibility that these defective tRNAs titrate out an inhibitor of import.

tRNA maturation is a multistep process that, in vertebrates, takes place in the nucleus, and mature tRNAs are then exported into the cytoplasm [39,40]. Export of tRNA by exportin-t, for example, requires the tRNA to be fully processed, providing a means to proofread tRNAs before they leave the nucleus [31,32,41]. Therefore it is not obvious why some tRNA species should be actively re-uptaken into the nucleus in human cells. One possibility could be that, given their relatively long half-life, some mature tRNAs are damaged in the cytoplasm by nucleases that remove one or more nucleotides from the 3′ CCA end. The resulting tRNAs cannot be aminoacylated, but may interfere with protein translation. This would then require the rapid removal of the defective tRNAs either by repairing the 3′ CCA end in the cytoplasm or by sequestering them into the nucleus where they might be repaired or degraded. There is evidence in yeast cells that tRNAs lacking the 3′ CCA end do accumulate and cause a growth defect if repairing enzymes are depleted from the cytosol [42]. Thus, nuclear re-uptake of tRNAs with a defective CCA end would provide a quality control system in addition to nuclear export. An alternative way to explain preferential accumulation into the nucleus of tRNAs lacking the full 3′ CCA end might be that mature tRNAs are quickly imported and then re-exported in the cytoplasm in our assay. Moreover, in permeabilized cells, mature tRNAs may bind to cellular factors, which may retain them in the cytoplasm, making them unsuitable substrates for nuclear import. Interestingly, the G3 T mutant with a 3′ CCA end lost most of its dominant negative phenotype, suggesting that it might be sequestered by cellular factors different from those involved in nuclear import. Thus, on the basis of the present data, we cannot exclude that mature tRNAs are also actively imported into the nucleus of human cells. Future work will address this important aspect.

It is long recognized that cellular tRNAs are packaged into retroviral particles with a stoichiometry of 20 or more molecules per virion [35] and that some of them have defective 3′ ends [43]. About 10% of these tRNAs bind tightly to the viral genome and serve as primers for reverse transcription, but the role of the other, less tightly bound tRNAs is unknown. Remarkably, in several cases, virus incorporation of tRNAs is selective. For example, avian retroviruses preferentially package tRNA^Trp^, and MLV packages tRNA^Pro^ and all lentiviruses in addition to mouse mammary tumour virus (MMTV), Foamy viruses (FV) and Mason-Pfizer monkey virus preferentially package tRNA^Lys^ [43–49]. tRNA incorporation into viral particles is mainly mediated by *gag* and reverse transcriptase, which is important for specific tRNA selection [50–52]. Our data suggest that some of these tRNA species may be important for nuclear import of retroviral genomes. Such tRNA species, by binding to the viral complex either before budding or perhaps after entry into the cell, may act as adaptors and allow RTC engagement with the cellular nuclear import machinery following virus uncoating [13,53].

Our results point to the existence of two or more cellular pathways mediating RTC nuclear import, one dependent on imp7 and another dependent on tRNAs. In this respect it may be relevant that nuclear export of mRNA and tRNA is also mediated by multiple and often redundant factors [40,54,55]. It will be interesting to investigate in more detail the interactions between these two pathways and whether cells with a low metabolism (like resting CD4^+^ memory T cells and resident macrophages) also accumulate defective tRNA species similar to starved yeast cells [56] and preferentially use the tRNA pathway for HIV-1 nuclear import.

## Materials and Methods

### Cell culture, recombinant virus production, and RTC purification.

HeLa and 293T cells were grown in Dulbecco's modified Eagle's Medium (DMEM) with Glutamax (GIBCO Labs, Paisley, United Kingdom) supplemented with 10% foetal calf serum (FCS) (Helena Bioscience, Newcastle, United Kingdom), SupT1 and Jurkat cells were grown in RPMI 1640 media (GIBCO) supplemented with 10% FCS at 37 °C in a humidified atmosphere containing 5% CO_2_. Human primary monocytes were isolated and grown as described previously [14]. HIV-1 vectors were made and purified as described previously [57] using plasmids pCSGW, expressing GFP; pCMVΔR8.2, expressing viral core proteins, and pMD.G, expressing VSV-G [58,59]. Moloney MLV was made using plasmid pCNCG, pGP NB, and pMD.G [60]. The MHIV-1 *gag* mutant was made using plasmid MHIV-mMA12CA-delEnv-luc and pHR′ (pMD.G was also used for infection experiments) [13]. RTCs were purified from acutely infected HeLa cells or 293T cells (for MLV) and labelled with YOYO-1 as previously described [14]. Cells (6 × 10^4^ well/24-well plates) were arrested in G1/S by treatment with 1.5-μg/ml aphidicolin (Sigma, St. Louis, Missouri, United States) for 24 h in complete media, infected, and analyzed by flow cytometry 24 h after infection.

### Preparation and initial fractionation of HSEs.

HSEs were prepared from HeLa, SupT1, and Jurkat cells as previously described [20]. Further fractionation was achieved by stepwise precipitation with saturated AS to final concentrations of 15%, 30%, 45%, and 60%. After addition of AS, samples were stirred at 4 °C for 1–2 h, centrifuged for 20 min at 6,000×*g* at 4 °C, and supernatants were used in the next round of precipitation. Pellets and supernatants were re-suspended in import buffer (20 mM HEPES-KOH [pH 7.3], 110 mM potassium acetate, 5 mM magnesium acetate, 0.5 mM EGTA) and dialyzed overnight against multiple changes of the same buffer. Fractions were normalized for protein concentration, tested in the nuclear import assay, and analyzed by SDS-PAGE. The 60P was depleted of importins by addition of 3 mM final β-mercaptoethanol and loading the sample onto a 1-ml low-substitution Phenyl-Sepharose column (Amersham, Little Chalfont, United Kingdom) equilibrated with import buffer supplemented with 250 mM sucrose and 3 mM β-mercaptoethanol at a flow rate 0.15 ml/min. The flow-through fraction was depleted 60P.

### Isolation of the active fraction from HSE.

The active fraction was isolated from HSE in the following steps. A saturated solution of AS was added to HSE to a final concentration of 60%. The sample was stirred at 4 °C for 1–2 h and centrifuged for 20 min at 6,000×*g*. The 60S was collected and applied to a 5-ml high-substitution Phenyl-Sepharose column (Amersham) equilibrated with buffer HDM (50 mM HEPES [pH 7.3], 1 mM DTT, 1 mM EDTA) + 60% AS at a flow rate of 1 ml/min. The column was washed with 25 ml of HDM + 60% AS and eluted with 25 ml of 60% to 0% AS gradient in HDM. Peak fractions (280 nm) were collected and dialyzed overnight against 5 l of 20 mM HEPES (pH 7.3) buffer and concentrated by centrifugation in Centricon YM-3 (Millipore, Billerica, Massachusetts, United States). Samples were further fractionated in a Mono Q PC 1.6/5 column (Amersham) equilibrated in buffer A (20 mM Tris-HCl [pH 7.6]) at a flow rate of 1 ml/min. The column was washed with five column volumes of buffer A and eluted with 15 column volumes of a 0 to 1 M NaCl linear gradient in buffer A. Fractions (0.1 ml) were collected, and peak fractions pooled, dialyzed overnight against 4 L of import buffer, and concentrated by centrifugation in Centricon YM-3. After normalization for protein concentration, fractions were tested in the nuclear import assay and analyzed by SDS-PAGE. To obtain the 60S NA, the concentrated active fraction after chromatography on Phenyl-Sepharose column (from HeLa, SupT1, and Jurkat cells) was treated with Proteinase K (10 μg/ml) at 37 °C, 30 min, and nucleic acids were extracted by phenol/chloroform and ethanol-precipitated.

### 3′-end labelling by T4 RNA ligase.

60S NA, total RNA from HeLa cells, and 21mer siRNA were dephosphorylated (90-μl reaction, 37 °C, 30 min, 45-U Shrimp Alkaline Phosphatase [Roche, Basel, Switzerland]). The reaction was stopped by phenol/chloroform extraction, and ethanol-precipitated RNA was then 3′-end radiolabelled (10-μl reaction, 37 °C, 30 min, 20 mM Tris-HCl [pH 7.8], 10 mM MgCl_2_, 10 mM DTT, 1 mM ATP, 15% DMSO, 26-U T4 RNA ligase [New England Biolabs, Beverly, Massachusetts, United States], 10-μCi 5′-[^32^P]pCp). The reaction was stopped by addition of an equal volume of StopMix (8 M urea/50 mM EDTA) and directly loaded onto a 15% denaturing PAGE. 5′-end radiolabelled (by standard procedure recommended for T4 polynucleotide kinase) pBP322DNA-MspI (New England Biolabs) and oligonucleotide sizing markers (range, 8–32 bases) (Amersham) were used as standards. After electrophoresis, the gel was dried and visualised by Storm 860 PhosphoImager (Molecular Dynamics, Sunnyvale, California, United States).

### Size fractionation of 60S NA and isolation of fraction 2.

The 60S NA was separated by 15% denaturing PAGE and visualized by ethidium bromide (Sigma) or SYBR Gold (Molecular Probes, Eugene, Oregon, United States) staining according to the manufacturer's instructions. Nine bands of different sizes were excised from the gel and eluted overnight at 4 °C into 0.5× TBE, 0.3 M NaCl buffer. The RNA was recovered by ethanol precipitation in the presence of 20-μg/ml glycogen (Roche). All samples were re-suspended in RNAse-free import buffer, normalized for RNA concentration, and tested in the nuclear import assay.

### Cloning of the small RNA molecules.

The small RNAs contained in fraction 2 (Fr2) were cloned using a method modified from Elbashir et al. [22]. A total of 5 μg of Fr2 was dephosphorylated (130-μl reaction, 37 °C, 30 min, 75-U Shrimp Alkaline Phosphatase [Roche]). The reaction was stopped by phenol/chloroform extraction, and RNA was ethanol precipitated. The 3′ adaptor oligonucleotide (pUUUaaccgcatccttctcx: where uppercase indicates RNA, lowercase indicates DNA, p represents phosphate, and x represents 4-hydroxymethylbenzyl) (Dharmacon, Lafayette, Colorado, United States) was ligated to 1 μg of dephosphorylated Fr2 (15-μl reaction, 37 °C, 30 min, 5 μM 3′ adapter, 50 mM Tris-HCl [pH 7.6], 10 mM MgCl_2_, 0.2 mM ATP, 0.1-mg/ml acetylated BSA, 15% DMSO, 25-U T4 RNA ligase [Amersham]). The ligation reaction was stopped by addition of an equal volume of StopMix and directly loaded onto a long (24 cm) 15% denaturing PAGE. pBP322DNA-MspI (New England Biolabs) and oligonucleotide sizing markers (range, 8–32 bases) (Amersham) were used as a size standard. After electrophoresis, the gel was stained with SYBR Gold (Molecular Probes). Ligation efficiency was approximately 50%. The ligation product was recovered from the gel and concentrated by ethanol precipitation. The ligation product was mixed with 100 pM 3′ RT primer (GACTAGCTGGAATTCAAGGATGCGGTTAAA) and heated 2 min at 90 °C. Reverse transcription (20-μl reaction, 42 °C, 1 h followed by 70 °C, 15 min, 200-U SuperScript II RNaseH^−^ reverse transcriptase (Invitrogen, Carlsbad, California, United States) in recommended buffer) was followed by RNAse H (Invitrogen) treatment. Synthesis of the first cDNA strand was monitored by a parallel RT reaction in the presence of [^32^P] dCTP. A poly C tail was added to the 3′ end of the first cDNA strand (20 μl reaction, 37 °C, 15 min, 25-U terminal transferase [New England Biolabs]), and the reaction stopped by heat inactivation at 75 °C, 10 min. Formation of the poly C tail was monitored by a parallel terminal transferase reaction in the presence of Cy5-dCTP. The terminal transferase product was PCR amplified (with *Pfu Taq* polymerase) using RACE-G primer (GACCACGCGTATCGATGTCGAGGGGGGGGGGGGGGGG (A, C, and T)) and the 3′ RT primer. The PCR products were gel purified by 7.5% non-denaturing PAGE, eluted, and ethanol precipitated. The PCR products were then directly ligated into pCR2.1-TOPO vector using TOPO TA cloning kit (Invitrogen). Colonies were screened by PCR and directly used for custom sequencing.

### Nuclear import assay.

Cells, approximately 50% confluent in gelatin-coated 5-mm diameter microdishes (MaTek Corp., Ashland, Massachusetts, United States), were washed in import buffer and permeabilized for 5 min on ice in import buffer containing 40-μg/ml digitonin (Fluka, Buchs, Switzerland). After two washes in import buffer, samples were incubated for 15 min at 37 °C in a tissue culture incubator in the presence of approximately 10^3^ labelled RTCs/cell, recombinant imp7 or small RNAs (concentrations indicated in figure legends), and an energy-regenerating system (1 mM ATP, 1 mM GTP, 20 mM creatine phosphate, 40-U/ml creatine phosphokinase) in 30-μl final volume. Samples were washed in import buffer, fixed on ice for 5 min with 2% paraformaldehyde in import buffer, and analyzed directly by confocal microscopy. Images acquired by confocal microscopy were analyzed by MetaMorph software version 4.5r4 (Universal Imaging Corp, Molecular Devices, Wokingham, United Kingdom) for quantification. Thresholding was performed to define areas to be analyzed. The threshold value was set so that no objects were visible in negative control samples. Integrated morphometric analysis (total grey value) was performed on the visible objects in the test samples, and the total grey value divided by the number of nuclei per field (Figure S2).

## Supporting Information

Figure S1 60S NA Purified from Jurkat and SupT1 T-Lymphocytic Cell Lines Stimulate RTC Nuclear Import (A) 15% denaturing PAGE of the 60S NA purified from the indicated cell lines followed by SYBR Gold staining (200 ng/lane). (B) Nuclear import of YOYO-1–labelled RTCs in permeabilized HeLa cells in the presence of 1× energy-regenerating system and 60S NA from the indicated cell line (100 ng) or buffer (ctr −). Scale bar indicates 25 μm.
(3.1 KB TIF)

Figure S2 MetaMorph Image Analysis for Quantification of RTC Nuclear Import Nuclear import was performed in permeabilized HeLa cells in the presence of fluorescent-labelled RTCs, 1× energy mix and buffer (ctr−) or 140-nm G3 RNA (test). Samples were fixed, then images were acquired by confocal microscopy and processed by Metamorph software version 4.5r4. The threshold signal of the starting image is adjusted to select all objects of interest (overlaid red cells) in both control and test samples (thresholding). Next, the image is “segmented” so that areas in which the pixel intensities are above the threshold range (red nucleoli and nuclear envelope) will be marked in the image (segmenting). Finally, the software integrates the signal from the areas shown in the segmentation images only (now shown in bright green and present mainly in the nucleoli) and gives the total grey value. This value is divided by the total number of nuclei in the field to give the final reading. Because only the bright green signal is integrated, the background cytoplasmic signal (darker green) is not included in the total grey value.
(6.6 KB TIF)

Figure S3 FISH Analysis following the Nuclear Import Assay FISH was performed with an RTC-specific probe following nuclear import in permeabilized HeLa cells in the presence of 1× energy-regenerating system (RTC + E) and 140 nM G3 RNA (RTC + E + G3) or 140 nM B2 RNA (RTC + E + B2). Control experiments in the absence of RTCs (CTR−) were performed to test the specificity of the probe and samples were analyzed by confocal microscopy. Scale bar indicates 12 μm.
(2.7 KB TIF)

Figure S4 The G3 RNA Does Not Promote Nuclear Import of MLV RTCs Nuclear import assay in permeabilized HeLa cells in the presence of 1× energy mix, approximately 10^5^ MLV or HIV-1 RTCs/assay and buffer (buffer) or 280 nM G3 (G3) or B2 RNAs (B2). Images were acquired by confocal microscopy using the same settings.
(2.5 KB TIF)

Figure S5 60S NA, Fraction 2, G3 RNA, and G3 T RNA Molecules Do Not Affect Nuclear Import of Maltose Binding Protein Fused to the M9 Peptide (M9) (A) Nuclear import assays were performed in permeabilized HeLa cells in the presence of 1× energy mix, 1 μM fluorescent-labelled M9 and 1 μM transportin (M9 + TR) or 100 ng 60S NA (M9 + 60S NA), 100 ng fraction 2 (M9 + Fr2), 140nM (corresponding to 100 ng) G3 RNA (M9 + G3), 140 nM B2 RNA (M9 + B2), 672 nM G3-T RNA (M9 + G3T), 672 nM G3-T RNA, and 1 μM transportin (M9 + TR + G3 T). (B) Nuclear import assays were performed in parallel with experiments shown in (A) in the presence of labelled RTCs and buffer (ctr−) or 140 nM G3 RNA. Note that the settings of the confocal microscope were different between (A) and (B) because M9 produced a stronger fluorescence signal than RTCs.
(956 KB TIF)

Figure S6 G3 T RNA, Lacking the D- and Anticodon Arms, Is a Dominant Negative Mutant (A) Quantification of RTC accumulation in HeLa nuclei following the import assay in the presence of 1× energy-regenerating system, 140 nM G3 RNA, and the mutant G3T RNA (with 3′ C end) at the concentrations indicated. Bars represent mean fluorescent value ± standard deviation of three independent experiments. (B) Same as (A), but the assay was performed in the presence of G3 T (filled triangles) and G3T + 3′ CCA end (empty triangles). Data are representative of two independent experiments. Images were acquired by confocal microscopy and analyzed by MetaMorph software version 4.5r4. Samples were threshold at the same values and the total fluorescence divided by the number of cells per field. Samples containing RTC + buffer were given an arbitrary value of 100%. At least 150 cells were counted per experiment.
(1.0 KB TIF)

Protocol S1 Supplementary Materials and Methods (33 KB DOC)

Table S1 Mutagenesis of Clones Contained in Fraction 2 (38 KB DOC)

Table S2 Primers Used for Generation of the T7 RNA-Polymerase Templates (55 KB DOC)

## Abbreviations

AS: ammonium sulphate
HSE: high-speed cytosolic extract
imp7: importin 7
MLV: murine leukaemia virus
NA: nucleic acids
RTC: reverse transcription complex
60P: pellet from 60% ammonium sulphate precipitation of high-speed cytosolic extracts
60S: supernatant from 60% ammonium sulphate precipitation of high-speed cytosolic extracts
