# Supplementary material for: tRNAs Promote Nuclear Import of HIV-1 Intracellular Reverse Transcription Complexes
Source: PLoS Biol. 2006 Oct 3;4(10):e332. doi: 10.1371/journal.pbio.0040332 (PMC1584419; doi:10.1371/journal.pbio.0040332)
Supplement: Supplementary file 1 [file pbio.0040332.sd001.doc]

**Supplementary Materials and Methods.**

**Generation of mutant clones and in vitro transcription of tRNAs.**

Mutant clones were obtained by PCR using mutagenic primers (see Supplementary Tables 1 and 2) with *Pfu* DNA Polymerase (Promega) except for the last extension cycle. PCR products were directly ligated into pCR2.1-TOPO vector and sequenced. Templates for *in vitro* transcription reactions were obtained by PCR amplification of pCR2.1-TOPO clones and their mutants using the corresponding 46 nt forward primers (containing the T7 RNA polymerase promoter and poly G site) and 21 nt reverse complementary primers (see supplementary Table 2) using *Pfu* DNA polymerase. To obtain templates for *in vitro* transcription of the G3 clone deletion mutants (G3 AnC-T, G3 D and G3 T), forward and reverse complementary DNA oligonucleotides, containing the T7 promoter, were purchased from Sigma-Genosys (see Supplementary Table 2). Double stranded DNA templates suitable for T7 transcription were obtained by annealing the two oligonucleotides at 95 ºC for 5 min in 40mM Tris-HCl (pH 8.0) buffer followed by cooling to 15 ºC at rate of 1 ºC/min and incubation on ice for 5 min. T7 transcription reactions (50 µl, 40mM Tris-HCl (pH 8.0), 22mM MgCl_2_, 1mM spermidine, 5mM DDT, 0.5% Triton X-100, 4mM of ATP, CTP, GTP and UTP, 20 U T7 RNA Polymerase-Plus (Ambion), 1µg purified PCR product) were incubated for 2h at 37ºC and 4U of DNAseI RNAse-free (Ambion) was added for 30 min at 37ºC. The reaction was stopped by addition of an equal volume of StopMix, directly loaded onto a 18x24x0.075cm 15 % denaturing PAGE and electrophoresis performed at 45^0^C constant 40W max for 5 hours. The gel was stained with ethidium bromide (Sigma) and T7 polymerase products of the right size were recovered from the gel and concentrated by ethanol precipitation.

**Preparation of viral nucleic acids.**

Approximately 120 ml of high-titre (≥10^7^ cfu/ml) virus stocks were filtered through a 0.45μm filter, incubated with 70U/ml DNAseI (Roche) (not RNAse free), 5mM MgCl_2_, for 1 h at 37^0^C and purified by ultracentrifugation through a 45-25% sucrose cushion in 50mM Na phosphate buffer (pH 7.4) at 23,000 rpm for 2h at 4^0^C in a Surespin rotor (Sorvall). The interface was diluted in 5 volumes of 50mM Na phosphate buffer (pH 7.4) and centrifuged through a 25% sucrose cushion as before. The pellet was re-suspended in 50μl PBSA + 2mM EDTA and analysed by electron microscopy. Purified virions were treated with 10 µg/ml proteinase K (Sigma) in 50mM Tris-HCl (pH 8.0), 0.4% SDS, 10mM EDTA for 2h at 55 ºC. The reaction was stopped by phenol/chloroform extraction and nucleic acids were ethanol precipitated. Total viral nucleic acids were re-suspended in import buffer, mixed with one volume of loading buffer (10mM Tris-HCl (pH 7.5), 90% formamide, 1% SDS, 0.1% bromphenol blue), heated at 90 ºC for 3 min and immediately loaded onto a 21x50x0.075cm 15 % denaturing PAGE. Electrophoresis was performed at 50^0^C constant 65W max for 7 hours in a Sequi-Gen GT nucleic acid electrophoresis cell (Biorad) and nucleic acids were visualized by SYBR Gold (Molecular Probes) staining. Bands having the size of interest were excised and eluted overnight at 4ºC into 0.5 x TBE, 0.3M NaCl buffer and recovered by ethanol precipitation in presence of 20 µg/ml glycogen (Roche). Samples were re-suspended in import buffer, normalized for RNA concentration and tested in the nuclear import assay.

# SDS-PAGE for protein and nucleic acids separation and Western blot.

Proteins were separated using either denaturing discontinuous gel electrophoresis (SDS-PAGE) (61) or TrisHCl non-denaturing polyacrilamide gels with a pH range of 7.1 to 8.9 (native PAGE). For Western blot, proteins were transferred onto PVDF membranes and the membrane probed using anti-importin 7 and anti-Ran antibodies as previously described (Fassati et al. 2003). Nucleic acids were separated in 15 % denaturing polyacrylamide gels (15% (wt/vol) acrylamide, 0.3% (wt/vol) N,N’- methylenbisacrylamide , 7M urea in 89mM Tris base/89mM boric acid/2mM EDTA. Gel runs were performed in 0.5 x TBE on the basic slab system (18 x 24 x 0.075cm gels) (Amersham) after 1h pre-run for 3.5h at 45 ºC constant for 5 hours at 40W max or on the Sequi-Gen GT nucleic acid electrophoresis cell (21 x 50 cm gels) (Bio-Rad) after 2h pre-run for 7 hours at 50 ºC constant 65W max. Gel temperature was controlled using a PowerPac 3000 with temperature probe (Bio-Rad).

**Northern Blot**

RNA was separated by 15% denaturing PAGE, visualized by SYBR Gold (Molecular Probes) staining and transferred to a nylon membrane (BrightStar-Plus Nylon membrane, Ambion) by electroblotting in a SemiPhore (Hoefer) semi-dry apparatus at 200 mA constant for 2 hours. RNA was UV crosslinked to the membrane using a crosslinker device (BDH), 120 mJ burst over 30 seconds. A specific probe to tRNA^Lys1,2^ was generated by 5’-end labelling of the antisense DNA oligonucleotide (5’-CGCCCAACGTGGGGCTCG-3’) designed to hybridize to G2/G3 RNA (tRNA^Lys1,2^) (modified from 49). After 5’-end labelling (10 μl reaction, 37º C, 1 hour, 20pM antisense DNA, 20 pM [γ-^32^P] ATP (3000 Ci/mM), 1x Kinase Buffer (Promega), 10U T4 polynucleotide kinase (Promega)) the probe was directly used without any purification. Prehybridization, hybridization and washing of the membrane were performed according to the manufacturer’s instructions. After the final wash the membrane was exposed to a PhosphoImager screen and visualized by Storm 860 (Molecular Dynamics).

**Splint labelling of tRNA.**

1 μg of RNA was mixed with 30 pM of an oligonucleotide complementary to the 3’ end of G2 RNA but without the CCA tail (GTTTTTTTTCGCCCAACGTGGGGCTCG) or the same oligonucleotide with the CCA tail (GTTTTTTTTGGCGCCCAACGTGGGGCTCG) and incubated for 2 minutes at 85º C in 50mM Tris-HCl (pH 7.8), 10 mM MgCl_2_ and 1mM DTT. The annealing reaction was quenched for 30 minutes on ice, 5 µCi of [α-^32^P]dATP (3000Ci/mM) and 3.25 units of T7 DNA polymerase (Sequenase v. 2.0, Amersham) were added and the reaction was incubated for 1 hour at 37º C. The reaction was stopped by addition of an equal volume of StopMix and directly loaded on to a 18x24cm 15% denaturing PAGE. After electrophoresis the gel was dried and visualised by Storm 860 PhosphoImager (Molecular Dynamics).

# FISH

The probe was prepared by nick translation using plasmid pCSGW (59) and Alexa fluor 488-dUTP (Molecular Probes) following the manufacturer’s instructions. The nuclear import assay was performed on HeLa cells as described on poly-L-lysine-coated dishes. Cells were fixed in 4% paraformaldehyde in import buffer for 5 minutes on ice and washed in PBS. Following a quenching step with 0.5mM ammonium chloride for 5 minutes at room temperature, cells were treated with 0.4% Triton-X in PBS for 5 minutes at room temperature, DNA denatured by treatment with NaOH (pH 13.4) for 2 minutes followed by several washes in ice-cold PBS. The heat-denatured probe was incubated as described (Roldan et al. 2005. Nat Immunol. 2005 Jan; 6(1):31-41) with cells for 16 hours at 37^0^C in a tissue culture incubator followed by one wash in 4xSSC at room temperature and two washes in 2xSSC for 30 minutes each at 37^0^C. Cells were analyzed by confocal microscopy after a final wash in PBS.
