# Supplementary material for: tRNAs Promote Nuclear Import of HIV-1 Intracellular Reverse Transcription Complexes
Source: PLoS Biol. 2006 Oct 3;4(10):e332. doi: 10.1371/journal.pbio.0040332 (PMC1584419; doi:10.1371/journal.pbio.0040332)
Supplement: Supplementary file 2 [file pbio.0040332.st001.doc]

| **Supplementary Table 1***. Mutagenesis of clones contained in Fraction 2* | | |  |  | |  |  |
| --- | --- | --- | --- | --- | --- | --- | --- |
| Mutation | Mutant name | Template clone | | | Forward primer 5’-3’ | | Reverse complementary primer 5’-3’ |
| Disruption of T stem C62→G62 | G3 C62G | G3 | | | CCCGGCTAGCTCAGTCGGTA | | GCGCCCAACGTG**C**GGCACGA |
| Disruption of T stem C62→G62 + A64→C64 | G3 C62G + A64C | G3 | | | CCCGGCTAGCTCAGTCGGTA | | GCGCCCAACG**G**G**C**GGCACGA |
| Disruption of T loop- D loop pairing C56→U62 | G3 C56U | G3 | | | CCCGGCTAGCTCAGTCGGTA | | GCGCCCAACGTGGGGCAC**A**AACCCA |
| Shortened Acceptor stem | G2 nAS | G2 | | | GGGTAGCTCAGTCGGTAGAGC | | GGGGTGGGGCTCGAACCCACGAC |
| tRNA ^Lys 1,2^ cDNA sequence | tRNA ^Lys 1,2^ cDNA | G2 | | | **GCCCGG**CTAGCTCAGTCGGTAGAGCAT | | CGCCCAACGTGGGGCTCGAA |
| tRNA ^Lys 1,2^ cDNA sequence with addition of C tail at the 3’ end | tRNA ^Lys 1,2^ +C | tRNA ^Lys 1,2^ cDNA | | | GCCCGGCTAGCTCAGTCGGTAGAGCAT | | **G**CGCCCAACGTGGGGCTCGAA |
| tRNA ^Lys 1,2^ cDNA sequence with addition of CC tail at the 3’ end | tRNA ^Lys 1,2^ +CC | tRNA ^Lys 1,2^ cDNA | | | GCCCGGCTAGCTCAGTCGGTAGAGCAT | | **GG**CGCCCAACGTGGGGCTCGAA |
| tRNA ^Lys 1,2^ cDNA sequence with addition of CCA tail at the 3’ end | tRNA ^Lys 1,2^ +CCA | tRNA ^Lys 1,2^ cDNA | | | GCCCGGCTAGCTCAGTCGGTAGAGCAT | | **TGG**CGCCCAACGTGGGGCTCGAA |
| tRNA ^Lys 1,2^ 74nt | tRNA ^Lys 1,2^ size +C | tRNA ^Lys 1,2^ cDNA | | | CGGCTAGCTCAGTCGGTAGAGCAT | | **G**CGCCCAACGTGGGGCTCGAA |
| tRNA ^Lys 1,2^ 75nt | tRNA ^Lys 1,2^ size +CC | tRNA ^Lys 1,2^ cDNA | | | CGGCTAGCTCAGTCGGTAGAGCAT | | **GG**CGCCCAACGTGGGGCTCGAA |
| tRNA ^Lys 1,2^ 76nt | tRNA ^Lys 1,2^ size +CCA | tRNA ^Lys 1,2^ cDNA | | | CGGCTAGCTCAGTCGGTAGAGCAT | | **TGG**CGCCCAACGTGGGGCTCGAA |

Specific changes/additions to the sequence highlighted in red
