# Supplementary material for: tRNAs Promote Nuclear Import of HIV-1 Intracellular Reverse Transcription Complexes
Source: PLoS Biol. 2006 Oct 3;4(10):e332. doi: 10.1371/journal.pbio.0040332 (PMC1584419; doi:10.1371/journal.pbio.0040332)
Supplement: Supplementary file 3 [file pbio.0040332.st002.doc]

| **Supplementary Table 2**. *Primers used for generation of the T7 RNA-polymerase templates*. | |  | |  |
| --- | --- | --- | --- | --- |
| Clone/mutant | Forward primers 5’- 3’ | | Reverse complementary primers 5’ – 3’ | |
| A2 | *AGCTTAATACGACTCACTATAGGG*ATTGTGGTTCAGTGGTAGAAT | | GTGCATTGGCCGGGAATCGA | |
| A3 | *AGCTTAATACGACTCACTATAGGG*ATTGGTGGTTCAGTGGTAGAA | | GTGCATTGGCCGGGAAACGA | |
| A5 | *AGCTTAATACGACTCACTATAGGG*ATGGGTGGTTCAGTGGTAGAA | | GTGCATGGGCCGGGAAACGA | |
| A7 | *AGCTTAATACGACTCACTATAGGG*CGTTGGTGGTATAGTGGTGAG | | GGTGCGTTGGCCGGGAAACG | |
| A9 | *AGCTTAATACGACTCACTATAGGG*GTTGGTGGTATAGTGGTGAG | | GTGCGTTGGCCGGGAAACGA | |
| A11 | *AGCTTAATACGACTCACTATAGGG*GTTGGTGGTATAGTGGTTAG | | GTGCGTTGGCCGGGAATCGA | |
| B1 | *AGCTTAATACGACTCACTATAGGG*CCCTGTGGTCTAGTGGTTAG | | GTTCCCTGACCGGGAATCGA | |
| B2 | *AGCTTAATACGACTCACTATAGGG*CCCTGGTGGTCTAGTGGTTA | | GTAGCCTGACCGGGAAACGA | |
| B3 | *AGCTTAATACGACTCACTATAGGG*TCCCTGGTGGTCTAGTGGTTA | | GTGCCCTGACCGGGAAACGA | |
| B4 | *AGCTTAATACGACTCACTATAGGG*CACATTGTCTAGCGGTTAGG | | GTTCCCACACCGGGAGTCGA | |
| G1 | *AGCTTAATACGACTCACTATAGGG*CCGGCTAGCTCAGTCGGTAG | | GGCGCCCAACGTGGGGCCCG | |
| G2 | *AGCTTAATACGACTCACTATAGGG*CTAGCTCAGTCGGTAGAGCAT | | GGCGCCCAACGTGGGGCTCG | |
| G3 | *AGCTTAATACGACTCACTATAGGG*CCCGGCTACCTCAGTCGGTAG | | GCGCCCAACGTGGGGCACGA | |
| G4 | *AGCTTAATACGACTCACTATAGGG*CCCGGCTAGCTCAGTCGGTA | | GCGCCCAACGTGGGGCACGA | |
| G3 C62G | *AGCTTAATACGACTCACTATAGGG*CCCGGCTACCTCAGTCGGTAG | | GCGCCCAACGTGCGGCACGA | |
| G3 C62G + A64C | *AGCTTAATACGACTCACTATAGGG*CCCGGCTACCTCAGTCGGTAG | | GCGCCCAACGGGCGGCACGA | |
| G3 C56U | *AGCTTAATACGACTCACTATAGGG*CCCGGCTACCTCAGTCGGTAG | | GCGCCCAACGTGGGGCACAAACCCA | |
| G3 D-AnC | *AGCTTAATACGACTCACTATAGGG*CCCGGCTACCTCAGTCGGTAGAGCATGAGACTCTTAATCTCAGGGTC | | GACCCTGAGATTAAGAGTCTCATGCTCTACCGACTGAGGTAGCCGG*CCCTATAGTGAGTCGTATTAAGCT* | |
| G3 AnC-T | *AGCTTAATACGACTCACTATAGGG*TGAGACTCTTAATCTCAGGGTCGTGGGTTCGTGCCCCACGTTGG | | GCGCCCAACGTGGGCACGAACCCACGACCCTGAGATTAAGAGTCTCA*CCCTATAGTGAGTCGTATTAAGCT* | |
| G3 D | *AGCTTAATACGACTCACTATAGGG*CCCGGCTACCTCAGTCGGTAGAGCA | | TGCTCTACCGACTGAGGTAGCCGG*CCCTATAGTGAGTCGTATTAAGCT* | |
| G3 AnC | *AGCTTAATACGACTCACTATAGGG*TGAGACTCTTAATCTCAGGGTC | | GACCCTGAGATTAAGAGTCTCA*CCCTATAGTGAGTCGTATTAAGCT* | |
| G3 T | *AGCTTAATACGACTCACTATAGGG*TGGGTTCGTGCCCCACGTTGGGCGC | | GCGCCCAACGTGGGGCACGAACCCA*CCCTATAGTGAGTCGTATTAAGCT* | |
| G3 T +CCA | *AGCTTAATACGACTCACTATAGGG*TGGGTTCGTGCCCCACGTTGGGCGCCA | | TGGCGCCCAACGTGGGGCACGAACCCA*CCCTATAGTGAGTCGTATTAAGCT* | |
| G2 nAS | *AGCTTAATACGACTCACTATAGGG*TAGCTCAGTCGGTAGAGC | | GGGGTGGGGCTCGAACCCACGAC | |
| tRNA^Lys1,2^ cDNA | *AGCTTAATACGACTCACTATAGGG*CCCGGCTAGCTCAGTCGGTA | | CGCCCAACGTGGGGCTCGAA | |
| tRNA^Lys1,2^ +C | *AGCTTAATACGACTCACTATAGGG*CCCGGCTAGCTCAGTCGGTA | | **G**CGCCCAACGTGGGGCTCGAA | |
| tRNA^Lys1,2^ +CC | *AGCTTAATACGACTCACTATAGGG*CCCGGCTAGCTCAGTCGGTA | | **GG**CGCCCAACGTGGGGCTCGAA | |
| tRNA^Lys1,2^ +CCA | *AGCTTAATACGACTCACTATAGGG*CCCGGCTAGCTCAGTCGGTA | | **TGG**CGCCCAACGTGGGGCTCGAA | |
| tRNA^Lys1,2^  +C size | *AGCTTAATACGACTCACTATAGGG*CGGCTAGCTCAGTCGGTA | | **G**CGCCCAACGTGGGGCTCGAA | |
| tRNA^Lys1,2^ size +CC | *AGCTTAATACGACTCACTATAGGG*CGGCTAGCTCAGTCGGTA | | **GG**CGCCCAACGTGGGGCTCGAA | |
| tRNA^Lys1,2^ size +CCA | *AGCTTAATACGACTCACTATAGGG*CGGCTAGCTCAGTCGGTA | | **TGG**CGCCCAACGTGGGGCTCGAA | |

Specific additions to the sequence highlighted in red, T7 RNA-polymerase promoter highlighted in blue
